# Interfacial Model and Characterization for Nanoscale ReB_2_/TaN Multilayers at Desired Modulation Period and Ratios: First-Principles Calculations and Experimental Investigations

**DOI:** 10.3390/nano8060421

**Published:** 2018-06-10

**Authors:** Shangxiao Jin, Dejun Li

**Affiliations:** 1 College of Physics and Materials Science, Tianjin Normal University, Tianjin 300387, China; jinshangxiao@126.com; 2 Tianjin International Joint Research Centre of Surface Technology for Energy Storage Materials, Tianjin 300387, China

**Keywords:** ReB_2_/TaN multilayers, modulation structure, first-principles calculation, interfacial model, adsorption energy, interfacial energy

## Abstract

The interfacial structure of ReB_2_/TaN multilayers at varied modulation periods (*Λ*) and modulation ratios (*t*_ReB2_:*t*_TaN_) was investigated using key experiments combined with first-principles calculations. A maximum hardness of 38.7 GPa occurred at *Λ* = 10 nm and *t*_ReB2_:*t*_TaN_ = 1:1. The fine nanocrystalline structure with small grain sizes remained stable for individual layers at *Λ*= 10 nm and *t*_ReB2_:*t*_TaN_ = 1:1. The calculation of the interfacial structure model and interfacial energy was performed using the first principles to advance the in-depth understanding of the relationship between the mechanical properties, residual stresses, and the interfacial structure. The B-Ta interfacial configuration was calculated to have the highest adsorption energy and the lowest interfacial energy. The interfacial energy and adsorption energy at different *t*_ReB2_:*t*_TaN_ followed the same trend as that of the residual stress. The 9ReB_2_/21TaN interfacial structure in the B-Ta interfacial configuration was found to be the most stable interface in which the highest adsorption energy and the lowest interfacial energy were obtained. The chemical bonding between the neighboring B atom and the Ta atom in the interfaces showed both covalency and iconicity, which provided a theoretical interpretation of the relationship between the residual stress and the stable interfacial structure of the ReB_2_/TaN multilayer.

## 1. Introduction

Hard and wear-resistant coatings are increasingly used to reduce the material losses or to increase the lifetime of tools and machine parts. Thin-film structures consisting of alternating nanoscale multilayers have been attractive subjects in the area of protective coatings due to their extraordinary properties, such as their enormous hardness, which cannot be obtained in uniform bulk materials or in monolithic coatings of the constituent materials. These multilayers are usually produced with physical vapor deposition (PVD), e.g., reactive magnetron sputtering [1]. Recently, the superhardness of rhenium diboride (ReB_2_) has been discovered to possess a maximum hardness of 55.5 GPa [2]. Researchers developed this superhard material through the optimization of two parameters: the high valence electron density (Re has the second highest valence electron density of all the transition metals) and the bond covalency (B, C, and N form the strongest covalent bonds). The obtained ReB_2_ films were found to be superhard, as the intrinsic film hardness value (52 GPa) was close to that of bulk ReB_2_ [3]. Transition-metal diborides, such as TiB_2_, have also been studied extensively due to their high hardness and thermochemical stability [4,5]. In this paper, ReB_2_ is introduced as a multilayer system and is combined with tantalum nitride (TaN) to synthetize ReB_2_/TaN multilayers. We choose the TaN layer as a component of the multilayers because TaN and Ta have been extensively used in coating systems due to their good diffusion barrier properties and their relatively stable structure [6,7,8,9,10,11,12]. In addition, TaN is effective in forming nano-crystalline characteristics in multilayers [13,14,15,16]. In our previous work, we performed a preliminary study of a ReB_2_/TaN multilayer coating. It was found that the modulation period can control the mechanical properties of the ReB_2_/TaN multilayers and induce the highest hardness of 28 GPa and modulus of 345.9 GPa with a lower residual stress [17]. However, no simulation and calculation results of the interfacial model and hard mechanism were studied in order to advance the in-depth understanding of the relationship between the mechanical properties and the multilayered structure.

In most multilayers, for example, TiN/VN and Cu/Ni [18,19], the individual layers have the same crystal structure. In this work, therefore, TaN is chosen to achieve an isostructure with ReB_2_. Theoretical works suggest that three kinds of models have been used for the strengthening mechanism in multilayers. The first is the analytical model that is based on the Hall–Petch powder law *σ*~*Λ*^1/2^, where *Λ* is the modulated period thickness [19,20,21,22,23]. The strength/hardness versus the period fits to this law at nanometer length scales, since the dislocation pileups can be treated as a continuum. At the sub-micrometer length scale, too few dislocations reside in the pile-ups to be treated as a continuum, and the Hall–Petch relation must be modified. However, the dislocation pile-up-based models are incapable of describing the strength behavior at decreasing layer thicknesses at a few of tens of nanometers. The second type of model is based on the confined layer slip mechanism that involves the glide of the single Orowan-type loops that are bounded by two interfaces [24,25,26]. This builds on earlier works of a similar confined layer slip mechanism in plastic yielding thin films on substrates. The third kind of model involves atomistic simulations of dislocation transmission across interfaces that provide an upper bound estimation of the interface barrier strength. In our previous work, we also conducted a preliminary theoretical study of ReB_2_/TaN multilayers, in which only the interface of the TaN(100)/ReB_2_(001) was chosen to calculate the interfacial structure, which showed that the B-N interface had a stronger covalent bonding [27]. 

The aforementioned investigations focus exclusively on the dislocation generation and motion mechanisms. However, an additional complication for isostructural materials, which is also typically miscible, is that the interface broadening during deposition can decrease the hardness, making the interpretation of the hardness data more difficult [28]. The miscible, isostructural nanolayers that are discussed above are known to interdiffuse, and thereby to lose their enhanced hardness. Along with atomistic simulations of the interface, this paper presents new data and more in-depth interpretation on the hardness enhancement of ReB_2_/TaN multilayers.

In this work, in addition to studying the experimental changes in the mechanical properties, modulation period, and modulation ratio, we particularly focused on the hardness mechanism and the relationship between the mechanical properties, residual stress, and interfacial structure using first-principles calculations that are based on density functional theory (DFT). For the first time (to the authors knowledge), we calculated and found the contribution of the interfacial energy, adsorption energy, and the interfacial stability to the residual stress release by controlling the appropriate interfacial structure.

## 2. Materials and Methods

### 2.1. Experimental Methods

To evaluate the influence of the layer thickness on the results, two series of ReB_2_/TaN multilayers at different bilayers (modulation period, *Λ* and modulation ratio, *t*_ReB2_:*t*_TaN_) as well as monolithic coatings were synthesized using an Radio frequency (RF) magnetron sputtering system, which has been described previously in detail [29]. RF magnetron sputtering is a process that RF power is applied to magnetron sputter source to control sputter rate of the target. The biggest advantage of RF power supply is sputtering insulator targets, such as ReB_2_ and TaN. The substrates were deposited 7 cm away from two water-cooled magnetron sputter sources with 99.95% stoichiometric ReB_2_ and TaN targets in Ar (99.99%) at a total pressure of 0.5 Pa. Silicon (100) wafers were used as the substrate materials, which were chemically cleaned in an ultrasonic agitator in acetone and absolute alcohol, before being mounted in the vacuum chamber. Prior to deposition, the substrates were sputter cleaned for 10 min in pure argon plasma at −500 V. Different Λ and *t*_ReB2_:*t*_TaN_ were achieved by controlling a computer-driven shutter. To enhance the adhesion between the multilayers and the substrate, an approximately 30 nm-thick Ta buffer layer was deposited on the Si substrates, after which the TaN and ReB_2_ layers were deposited alternately. In the process of deposition, the sputtering power was 50 W for ReB_2_ and 110 W for TaN, and a negative bias of 80 V was applied to the substrate.

The cross-section of the sample was examined with a field-emission scanning electron microscopy (SEM, SU8010, Hitachi, Japan). X-ray diffraction (XRD) and X-ray reflection (XRR) scans were performed with Cu K_α_ (40 kV, 20 mA, *λ* = 1.54056 Å) radiation in a D8A diffractometer (Bruker, Karlsruhe, Germany). The scans were performed while keeping Χ and ψ fixed and varying *θ* at intervals of 0.02° (the symbols have their usual meanings [30]). The modulation period *Λ* was determined by matching the XRR peak positions. The layer thickness ratio was then adjusted so that the relative peak intensities matched. Nanoindentation and nanoscratch measurements on the as-deposited multilayers were performed at room temperature using a Nano indenter system (XP, Agilent, CA, USA). The hardness and elastic modulus of the multilayers as a continuous function of depth from a single indentation were obtained using the continuous stiffness measurement (CSM) technique in the nanoindentation test. The triangular Berkovich diamond indenter tip was calibrated using fused silica [24,31]. Each sample was indented ten times, at a maximum load of 40 mN, which yielded typical maximum depths of 400 nm. The maximum load in the scratch test was up to 100 mN in order to measure the fracture resistance. Then, a post-scan was performed to measure the profile of the scratch surface. The variation in the chemical composition and the element chemical bonding states of the multilayers were analyzed using X-ray photoelectron spectrometer (XPS, PHI 5300, Kanagawa, Japan).

### 2.2. Theoretical

To reveal the layer’s impact and hard mechanism in the multilayers, first-principles that was based on density functional theory (DFT) [32,33] was used to calculate the optimal electronic structure, according to the measured structure. The exchange correlation functional was treated using the generalized gradient approximation (GGA) with the Perdew–Burke–Ernzerhof (PBE) [34]. The interactions between ionic and valance electrons were described with the ultrasoft pseudopotential [35]. The *k*-point sampling and kinetic energy cutoff convergence have been tested for all calculated surfaces and interfaces. The theoretical calculation of all the surface and interfaces is done after the convergence test. The detailed theoretical calculation results of all the surface and interfaces will be given in the results and discussion section.

## 3. Results and Discussion

### 3.1. Microstructure Characterizations

**Figure 1:**
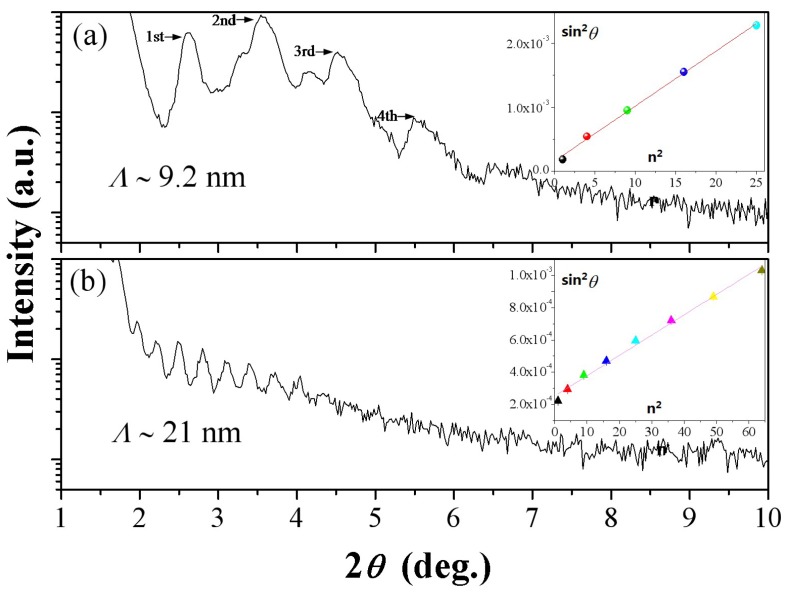
X-ray reflection (XRR) patterns of rhenium diboride (ReB_2_)/tantalum nitride (TaN) multilayers at (**a**) *Λ*~8 nm, *t*_ReB2_:*t*_TaN_ = 1:2; and (**b**) *Λ*~21 nm, *t*_ReB2_:*t*_TaN_ = 1:1. The insets are the linear least squares fit of sin^2^*θ* vs. *n*^2^.

The X-ray reflection (XRR) pattern that is shown in Figure 1a,b give the modulation information of the ReB_2_/TaN multilayers at two different modulation period (*Λ*), and it is used to accurately calculate *Λ* value. The reflection peaks of different orders *n* in the XRR spectra occur at 2*θ* positions given by the modified Bragg’s law [29]:

(1) Sin2θ=(nλ2Λ)2+2δ

where *λ* is the X-ray wavelength (1.5 Å), *δ* is related to the average reflective index, and *Λ* is the modulation period of a multilayer (bilayer period). A straight line is fitted according to spots of sin^2^*θ* vs. *n*^2^ to determine *δ* and *Λ*, while the error of each spot was determined using the internal *θ*. Using Formula (1), 9.2 and 21 nm-*Λ* are obtained from the linear regressions of the sin^2^*θ* versus *n*^2^ plots shown in Figure 1a and 1b, which are consistent with our design before the experiment. The strong multiple superlattice reflections that are resulting from the large difference in the X-ray scattering factors of the periodic layers indicate the existence of well-defined layered structures between two individual layers in the multilayer system, which are a beneficial characteristic for the hardness enhancement [24,29,31,36].

**Figure 2:**
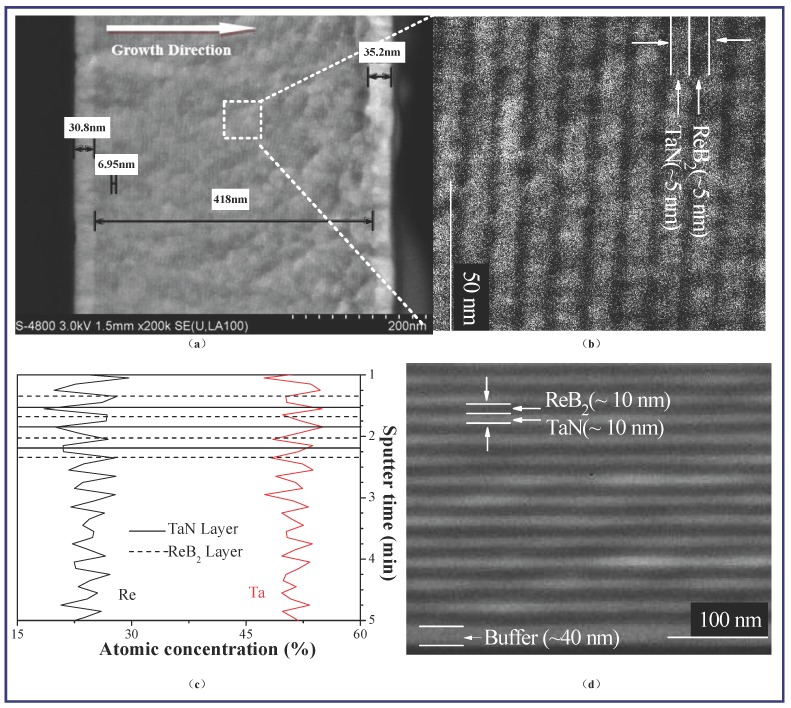
Cross-sectional scanning electron microscopy (SEM) images of ReB_2_/TaN multilayer at (**a**) *Λ*~7 nm at low magnification; (**b**) 7 nm, *t*_ReB2_:*t*_TaN_ = 1:2 at high magnification; (**c**) X-ray photoelectron spectrometer (XPS) depth profile of ReB_2_/TaN multilayer at *Λ*~7 nm, *t*_ReB2_:*t*_TaN_ = 1:2; (**d**) 20 nm, *t*_ReB2_:*t*_TaN_ = 1:1 at high magnification.

Figure 2a,b show the high-resolution cross-sectional scanning electron microscopy (SEM) images at low and high magnifications from identical ReB_2_/TaN samples. ReB_2_/TaN samples, as measured by the X-ray reflection (XRR), are 418 nm thickness. An approximately 31-nm-thick Ta buffer layer is observed. The coating replays the multilayered nanostructure at *Λ*~10 nm and clearly indicates planar interfaces along the growth direction. The observed 10-nm-thick *Λ* is near the calculated 9.2 nm, as shown in Figure 1.

The XPS depth profiles of this multilayer are shown in Figure 2c. The periodic variation of the concentrations of Re and Ta as the main elements throughout the thickness gives direct evidence of the multilayered modulation structure in our design. The formation of alternating ReB_2_ and TaN at the nanoscale is also confirmed. The elemental composition of Re:Ta = 1:2 within the bilayer thickness is nearly equal to the modulation ratio of *t*_ReB2_:*t*_TaN_ = 3.4:6.6, as observed in Figure 2b. Figure 2c is artificially corresponded with Figure 2b to suggest that Re shows a darker contrast. The typical cross-sectional SEM images of Figure 2b *Λ*~10 nm and Figure 2d *Λ*~20 nm are taken from identical ReB_2_/TaN samples that were used in the XRR measurements of Figure 1 and directly show a well-defined composition modulation and multilayered structure, which is in agreement with the XRR results above. The SEM results prove that the layered structures are defined well in broad nanolayers. The intermixing crossing interfaces can be observed in Figure 2a,b, due to the lack of sharp interfaces.

### 3.2. Mechanical Properties

**Figure 3:**
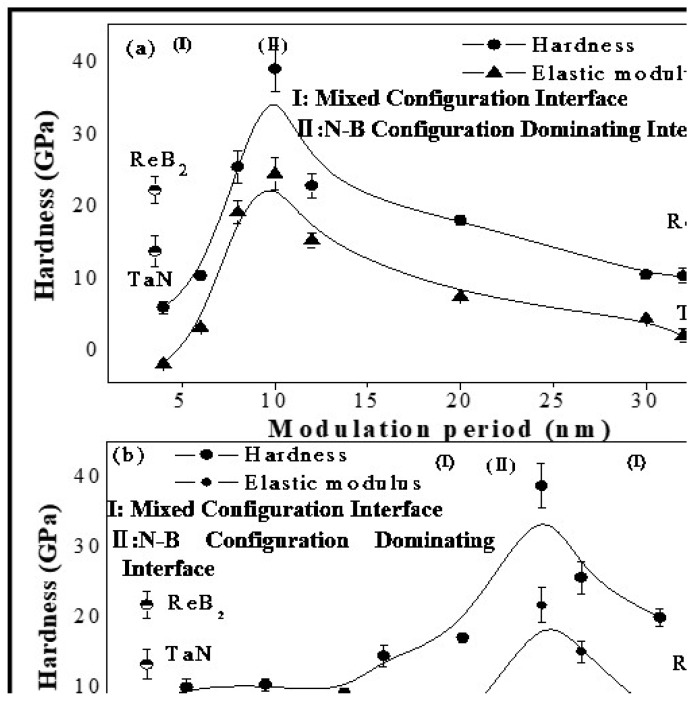
Hardness and Elastic modulus of ReB_2_/TaN multilayers vs. (**a**) *Λ*; (**b**) *t*_ReB2_:*t*_TaN_.

Figure 3 indicates the regularity of hardness and elastic modulus fluctuation versus *Λ* and *t*_ReB2_:*t*_TaN_ for the ReB_2_/TaN multilayers. To compare the multilayers, this figure also shows the hardness and elastic modulus values of monolithic ReB_2_ and TaN coatings synthesized under identical deposition conditions. The error bars are drawn using the standard deviation that was calculated from the 10 indents. As we can see, the results agree well with the change regulations of other types of the multilayers [6,13,14,15,16,17,18,19,23,31,36]. For the ReB_2_/TaN multilayers, the hardness reaches a maximum value of 38.7 GPa at *Λ* = 10 nm and *t*_ReB2_:*t*_TaN_ = 1:1, and then decreases as *Λ* increases or *t*_ReB2_:*t*_TaN_ decreases further.

The rule-of-mixtures hardness of the ReB_2_/TaN multilayers can be calculated using [18]:

(2) HReB2/TaN=HReB2tReB2Λ+HTaNtTaNΛ

The calculated rule-of-mixtures hardness of the ReB_2_/TaN multilayers at *Λ* = 10 nm and *t*_ReB2_:*t*_TaN_ = 1:1 is 17.6 GPa. Whereas, the measured hardness for this multilayer is up to 38.7 GPa, which is 120% higher than the rule-of-mixtures value. Detailed analyses of the deformation mechanisms and the interfacial model will be presented in Section 3.3.

Despite the high hardness, the performance of the multilayers depends on many other factors, including the residual stress, friction, fracture toughness, adhesive ability, etc. The reduction of residual stress in the multilayers is a key factor affecting their industrial applications. The residual stress σ is calculated applying the Stoney formula [37] and using the substrate curvature that is determined from a surface profiler:

(3) $$\sigma =-\frac{E_{s}t_{s}^{2}}{6t_{c}(1-v_{s}^{})R}$$

where *E*_s_ (131 GPa), *t*_s_ (0.0005 cm) and *v*_s_ (0.28) are, respectively, the elastic modulus, thickness, and Poisson’s ratio of the substrate; *t_c_* is the coating thickness; and, *R* is the radius of curvature of the multilayer coated substrate. In this test, we choose a scan length of 2 cm for the *R* measurement. Although, in our study, a precise residual stress is difficult to determine using the available instruments, and this result reflects the reducing trend of the residual stress when compared with the monolithic layer.

**Figure 4:**
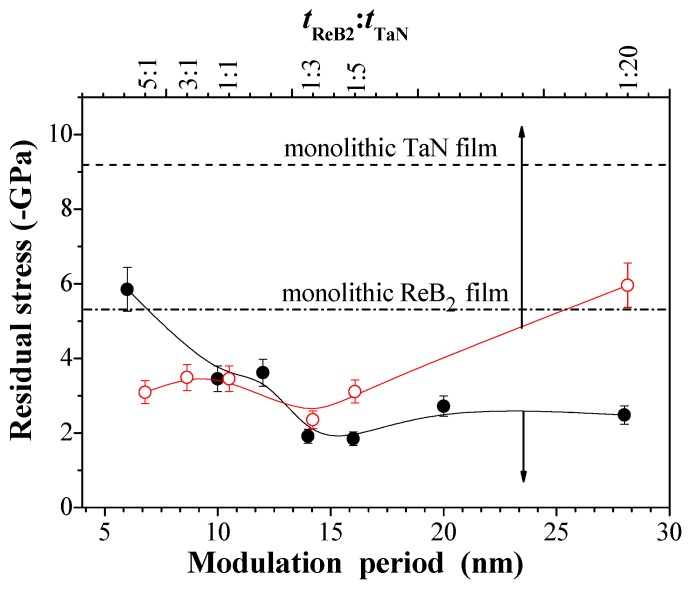
Residual stresses of ReB_2_/TaN multilayers vs. *Λ*, *t*_ReB2_:*t*_TaN_.

Figure 4 indicates the residual stress, i.e., compressive stress, at different *Λ* and *t*_ReB2_:*t*_TaN_ for the ReB_2_/TaN multilayers. To compare monolithic coatings with the multilayers, the residual stresses of individual ReB_2_ and TaN coatings that are synthesized under identical deposition conditions are also shown in this figure. The error bars are drawn using the standard deviation, as calculated from the 10 indents. Nearly all of the multilayers exhibit lower residual stress than the average value of the monolithic ReB_2_ and TaN coatings. The residual stress of the multilayers reaches the lowest value at *Λ*~10 nm and *t*_ReB2_:*t*_TaN_ = 1:3. We believe that periodic insertion of TaN into ReB_2_ layers suppresses the grain growth, which releases stress that is built up in the ReB_2_ layers.

The elastic modulus of the coatings is another important factor to obtain good wear resistance. The *H*^3^/*E*^2^ ratio is a strong indicator of the coating’s resistance to plastic deformation [38,39]. Table 1 listed these values of the multilayers with different parameters. The resistance plastic deformation of our hardest ReB_2_/TaN multilayer is obviously improved (*H*^3^/*E*^2^ = 25.8%) due to high hardness and a relatively low elastic modulus when compared with that of the other multilayers and monolithic ReB_2_ and TaN coatings. Note that another important advantage of the multilayer nanostructured coatings is that one can fabricate superhard materials with identical hardnesses but different values of the plastic deformation (*H*^3^/*E*^2^). This means that superhard multilayers can be produced with different combinations of elastic and plastic properties, which provides a wide choice of multilayers for various specific tasks. Thus, the service life of multilayers can be enhanced while using multilayers that better fit the application substrates, such as steel, in terms of minimizing the internal stresses that occur at the coating/substrate interface.

**Figure 5:**
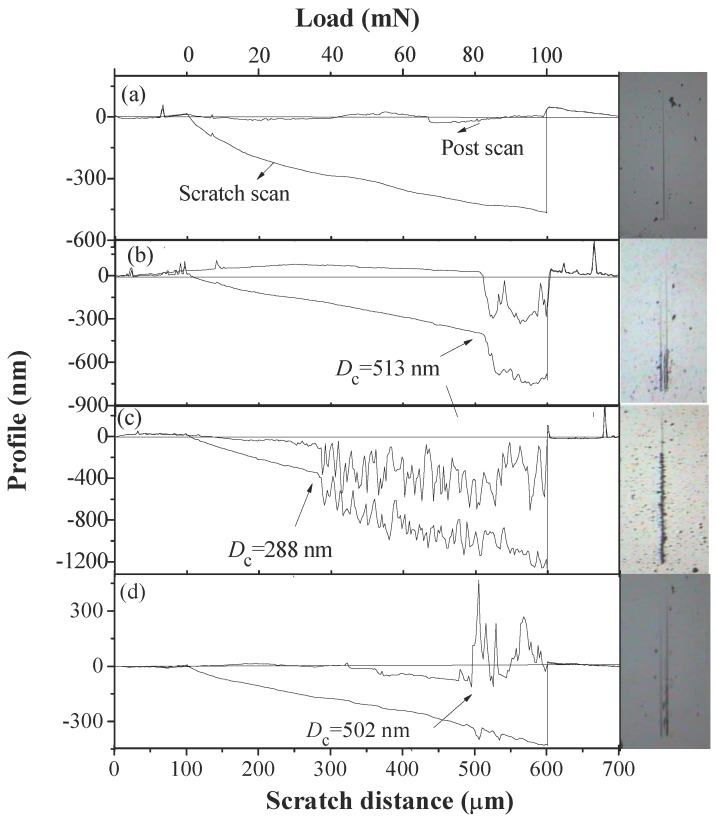
Surface profiles of the scratch scan, post scan, and scratch tracks on ReB_2_/TaN multilayers at different *t*_ZrB2_:*t*_AlN_ and *Λ*, (**a**) 1:1, 10 nm; (**b**) 1:4, 10 nm; (**c**) 2:1, 10 nm; and (**d**) 1:1, 28 nm.

Figure 5a-d show the results of the scratch test at different *t*_ZrB2_:*t*_AlN_ and *Λ*, reflecting the fracture resistance of the ReB_2_/TaN multilayers. The post-scan curves are always above the scratch scan curves in test due to the plastic recovery after scratching. The normal load corresponding to the point in which the scratch scan profile shows an abrupt change is the critical fracture load *L_c_* that can characterize the adhesion strength of the multilayers. The scratch scan profiles of all the multilayers indicate an abrupt increase point in the scratch depth, except the *t*_ReB2_:*t*_TaN_ = 1:1 and Λ = 10 nm sample. This means that the hardest multilayer at *t*_ReB2_:*t*_TaN_ = 1:1 and *Λ* = 10 nm has the highest fracture resistance and adhesion strength between the multilayer and the substrate, which fits the plastic deformation (*H*^3^*/E*^2^) results in Table 1 well. We believe that the improved fracture resistance appears to be directly related to a lower compressive stress, higher hardness, and strong plastic recovery of the coating with a multilayered structure.

### 3.3. Hard Mechanism

For the nano-indentations, the hardness is normally defined as the ratio of the maximum applied load divided by the corresponding projected contact area, i.e., H=PmaxAC, where *H*, *P*_max_, and *A_C_* are the hardness, the maximum applied load, and the projected contact area at the maximum applied load, respectively. However, in this case, several additional observations concerning the behavior of the supperlattice materials can be seen from the load versus displacement curves. This is done by examining different combinations of the curves of the two epitaxially grown ReB_2_/TaN multilayers and their monolithic components.

**Figure 6:**
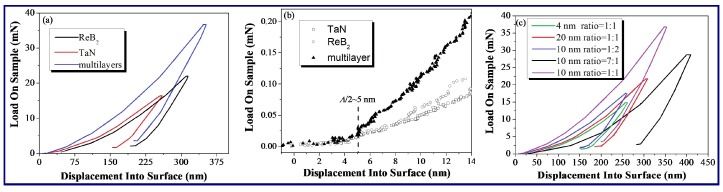
(**a**) Comparison of load *vs* displacement data of ReB_2_/TaN multilayer (*Λ*~10 nm) with monolithic ReB_2_ and TaN coatings; (**b**) Comparison of load vs. displacement data for ReB_2_/TaN multilayer (*Λ*~10 nm) with monolithic ReB_2_ and TaN coatings at shallow indentation depths; and, (**c**) Comparison of load vs. displacement data of ReB_2_/TaN multilayers at different *t*_ReB2_:*t*_TaN_ and *Λ*.

A comparison of the load versus displacement curves for ReB_2_, TaN, and their multilayer at shallow indentation depths and the resulting integral curves are shown in Figure 6a,b, respectively. When the same displacement is pressed into the surface, the multilayer requires a larger load than two monolithic coatings, indicating that the multilayer is harder than both of the monolithic coatings. Figure 6c shows the typical loading-unloading sequences for the multilayers. The highest hardness occurs at *Λ*~10 nm and *t*_ReB2_:*t*_TaN_ = 1:1, which agrees with the measured results that are mentioned above in Figure 3. The comparison in Figure 6b also indicates that ReB_2_ and TaN initially follow the same loading pattern as the multilayer, but start to deviate from it at approximately 5 nm. Since the coherent interface repeat periods for multilayers (*Λ*/2~5 nm) appear at 5 nm first, this phenomenon provides direct evidence that the behavior of the interface as a barrier to dislocation motion begins to affect the deformation of multilayer, leading to an increase in the hardness.

### 3.4. Theoretical Model, Calculation, and Discussion

To further explain the hardness mechanism and the relationship between the mechanical properties and the multilayered structure, we use first-principles that are based on density functional theory (DFT) to simulate the interfacial structure for which we must choose the appropriate interfacial configuration models to calculate and compare the interfacial energy, adsorption energy, charge density, and density of states (DOS).

To avoid the interactions between repeated slabs, a uniform vacuum width of 15 Å is employed while performing the calculations for all of the surfaces. The slabs are fully relaxed until the system energy is minimized. As a result, a plane wave cutoff energy of 280 eV and 300 eV is employed for the ReB_2_(001) and TaN(111) surfaces, which assures a total-energy convergence of 10^−5^ eV/atom. The Brillouin zone sampling is set with 6×6×1 and 5×5×1 Monkhorst-Pace *k*-point meshes for the ReB_2_(001) and TaN(111) surfaces, respectively. For the interfaces, the cutoff energy of the plane wave is chosen as 330 eV. Integrations in the Brillouin zone are performed while using the special *k*-points that are generated with 5×5×1 mesh grids.

**Figure 7:**
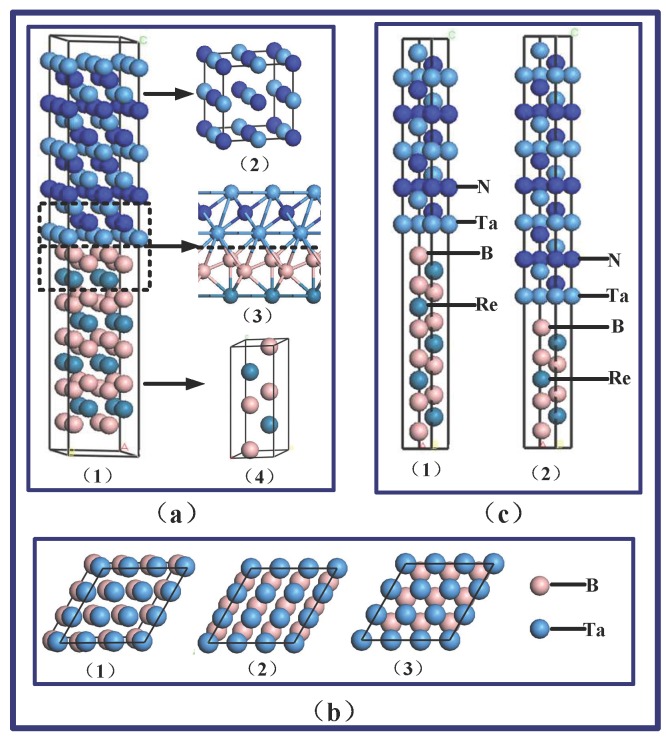
Supercell of BB-Ta interface (**a1**), crystal structures of TaN (**a2**) and ReB_2_ (**a4**) and the interface bands (**a3**); Structures of BB-Ta-top configuration (**b1**), BB-Ta-bridge configuration (**b2**) and BB-Ta-hcp configuration (**b3**), respectively; Unit cell structures of 15ReB_2_/15TaN interface (**c1**), and 9ReB_2_/21TaN interface.(**c2**).

In the initial calculation, we first have to choose some possible interfacial models to calculate some of the preliminary results. We obtain the surface of TaN(111) through the cutting of the TaN bulk (space group P-6M2) and the surface of ReB_2_ (001) through cutting the ReB_2_ bulk (space group P63/MMc). For the ReB_2_/TaN multilayers, six possible interfaces, including B-N, BB-N, Re-N, B-Ta, BB-Ta, and Re-Ta exist in the connection. The supercell, including 129 atoms of the BB-Ta interface and crystal structures of TaN and ReB_2_, can be seen in Figure 7(a). There are some different symmetry sites in each type of interface connection in Figure 7(a1-a4). We use both the N- and Ta-terminated sites of the TaN to simulate the ReB_2_(001)/TaN(111) interface and to choose three high-symmetry sites on which the interface atoms could bond. Using the B-N interface as an example, the B atoms could occupy the site on top of N atoms, which is called the B-N-Top configuration (Figure 7(b1)), or on the bridge site between two N atoms, which is called the B-N-bridge configuration (Figure 7(b2)). When there is a Ta atom in the second layer directly below the B atoms, we call it the hcp-hollow site, denoted as the B-N-hcp configuration (Figure 7(b3)). Eighteen different interface configurations of ReB_2_/TaN are chosen in the initial calculation. To simulate the interfaces of the multilayers, TaN and ReB_2_ slabs of thirteen layers are cut through using the CASTEP software. Each unit cell structure comprised of thirteen layers of TaN and thirteen layers of ReB_2_ is separated with a 15-Å-thick vacuum layer.

The adsorption energies (*E_ad_*) corresponding to eighteen interfacial models with different interfacial atoms are calculated from the energy difference per unit area corresponding to the introduction of the interface in comparison to the two separate slabs [40,41]:

(4) $$E_{ad}=\frac{E_{{\mathrm{ReB}}_{2}(001)}+E_{\mathrm{TaN}(111)}-E_{{\mathrm{ReB}}_{2}(001)/\mathrm{TaN}(111)}}{A}$$

where $E_{{\mathrm{ReB}}_{2}(001)/\mathrm{TaN}(111)}$ is the total energy of the ReB_2_/TaN, $E_{{\mathrm{ReB}}_{2}(001)}$ and $E_{\mathrm{TaN}(111)}$ are the total energies of the pure ReB_2_ and TaN interlayers with the TaN and ReB_2_ interlayers that are replaced by vacuum, respectively, in the same slab structure, and *A* is the area of the interface.

Table 2 lists the calculated *E_ad_* of the ReB_2_/TaN interface corresponding to eighteen different interfacial models. The general trend is that, the higher the adsorption energy, the stronger the chemical bonding at the interfaces. From Table 2, the obtained adsorption energy of the B-Ta interfaces is larger than that of other models, indicating that strength of this interfacial bonding is stronger than the others.

To provide a detailed explanation of the relationship between the residual stress and interfacial energy, adsorption energy, and interfacial structure, the ReB_2_(001)/TaN(111) interfacial configurations at five different thickness ratios. i.e., theoretical *t*_ReB2_:*t*_TaN_, are all built as B-Ta interfaces. When considering the ideal growth of the ReB_2_ and TaN layers, B-terminated surfaces of ReB_2_(001) and Ta-terminated surfaces of TaN(111) are built in the ReB_2_(001)/TaN(111) configuration for the B-Ta interfaces because they exhibit higher adsorption energies than the others. To avoid the influence of the layer number on the total energy, each supercell contains thirty layers of ReB_2_(001) or TaN(111). ReB_2_(001)/TaN(111) configurations at five different thickness ratios, namely, 3ReB_2_/27TaN, 9ReB_2_/21TaN, 15ReB_2_/15TaN, 21ReB_2_/3TaN, and 27ReB_2_/3TaN, are considered in the calculations. All the ReB_2_(001)/TaN(111) configurations at five different thickness ratios are B-Ta interfaces, each of which consists of a B-terminated ReB_2_(001) surface and a Ta-terminated TaN(111) surface. Figure 7(c1) and (c2) display the supercells of the 15ReB_2_/15TaN and 9ReB_2_/21TaN configurations.

The interfacial energy (*E_inter_*) shows how much weaker the interfacial bonding is, which is compared with the interlayer bonding in the bulk materials. The *E_inter_* is calculated while using the following equation:

(5) Einter=EReB2(001)/TaN(111)−NRenReERebulk−NBnB(EReB2bulk−ERebulk)−NTaETabulk−NN(ETaNbulk−ETabulk)A−EsurReB2(001)−EsurTaN(111)

where $E_{{\mathrm{ReB}}_{2}}^{bulk}$, ERebulk, $E_{\mathrm{TaN}}^{bulk}$, $E_{\mathrm{Ta}}^{bulk}$, $E_{{\mathrm{ReB}}_{2}(001)/\mathrm{TaN}(111)}$, $E_{sur}^{{\mathrm{ReB}}_{2}(001)}$, and $E_{sur}^{\mathrm{TaN}(111)}$ are the total energy of the relaxed ReB_2_ bulk material, Re bulk material, TaN bulk material, Ta bulk material, ReB_2_/TaN interface, the energy of ReB_2_(001) surface, and the TaN(111) surface, respectively; *N*_Re_, *N*_B_, *N*_Ta_, and *N*_N_ denote the number of Re, B, Ta, and N atoms in the ReB_2_/TaN interface, respectively; *n*_Re_ and *n*_B_ represent the number of Re atoms in the Re bulk material and that of B atoms in ReB_2_ bulk material, respectively; and, *A* is the interfacial area. Formula (5) is attributed to the nonstoichiometric nature in the calculations to eliminate the effect of spurious dipole interactions that might bias the results. The *E_sur_* that appears in Formula (5) is obtained from Formula (6),

(6) $$E_{sur}^{XY}=\frac{E_{slab}^{total}-\frac{N_{X}}{n_{X}}E_{X}^{bulk}-\frac{N_{Y}}{n_{Y}}(E_{XY}^{bulk}-E_{X}^{bulk})}{2A}$$

where $E_{slab}^{total}$, $E_{XY}^{bulk}$, and $E_{X}^{bulk}$ represent the total energies of a surface slab (ReB_2_ or TaN), ReB_2_ bulk (TaN bulk) and Re bulk (Ta bulk), respectively; $N_{X}$ and $N_{Y}$ denote the number of Re and B (Ta or N) atoms in the surface slabs, respectively; $n_{X}$ and $n_{Y}$ represent the number of Re atoms in Re bulk material (Ta atoms in Ta bulk material) and B atoms in ReB_2_ bulk material (N atoms in TaN bulk material), respectively; and, *A* is the corresponding surface area. Due to the nonstoichiometric symmetric slabs in Formula (6), the effect of spurious dipole interactions can be eliminated to ensure the calculation is accurate [42].

**Figure 8:**
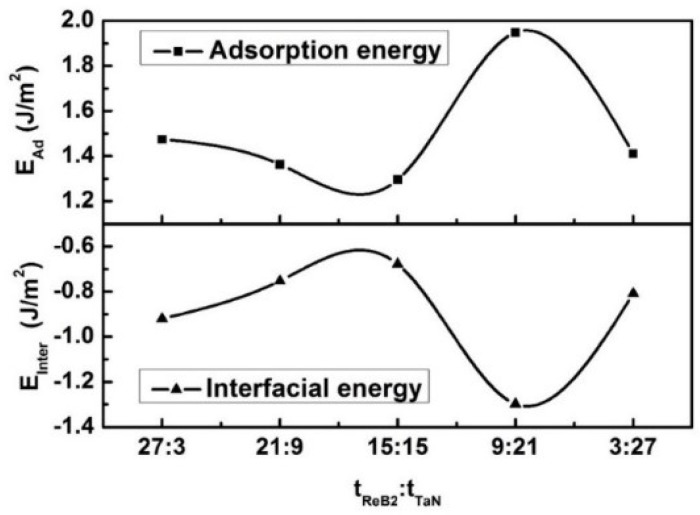
Adsorption energy (*E_ad_*) and interfacial energy of ReB_2_/TaN multilayers at different *t*_ReB2_:*t*_TaN_ based on five B-Ta interfacial models.

The calculated results involving adsorption energy and interfacial energy are shown in Figure 8 as a function of the theoretical *t*_ReB2_:*t*_TaN_ for the 3ReB_2_/27TaN, 9ReB_2_/21TaN, 15ReB_2_/15TaN, 21ReB_2_/3TaN, and 27ReB_2_/3TaN interfacial models. The higher the adsorption energy, the stronger the interfacial bonding and the lower the interfacial energy. Therefore, it can be clearly seen from Figure 8a,b that the change trend of adsorption energy is opposite to that of interface energy. The interfacial energy of a system measures the stability of the interface. The smaller the interfacial energy, the more stable the interfacial structure [43]. In particular, the interface will not form spontaneously when the interfacial energy of a system is higher than zero. According to Figure 8b, it is revealed that the ReB_2_(001)/TaN(111) interfacial structures at five different *t*_ReB2_:*t*_TaN_ that form spontaneously because the calculated interfacial energies of ReB_2_(001)/TaN(111) with five different models are negative. The interfacial energy of the 9ReB_2_/21TaN structure (−1.297 J/m^2^) is the lowest when compared with those of 27ReB_2_/3TaN, 21ReB_2_/3TaN, 15ReB_2_/15TaN, and 3ReB_2_/27TaN by −0.920 J/m^2^, −0.470 J/m^2^, −0.424 J/m^2^, and −0.505 J/m^2^, respectively, indicating that 9ReB_2_/21TaN is the most stable interfacial structure. This result is consistent with the adsorption energy. It is worth noting that the interfacial energy at different *t*_ReB2_:*t*_TaN_ follows the similar trend as that of the residual stress. A high residual stress easily causes some cracks in the coatings. Hence, the reduction of residual stress in the coatings is a key issue, which can improve their industrial applications. The interface with the 9ReB_2_/21TaN structure, whose theoretical *t*_ReB2_:*t*_TaN_ is experimentally close to 1:3, shows the highest adsorption energy and the lowest interfacial energy, meaning that it is the most stable interfacial bond. It is clear from Figure 4 that the multilayer at a *t*_ReB2_:*t*_TaN_ of 1:3 exhibits the lowest residual stress. We postulate that the low residual stress appears to be directly related to the stable interfacial bonding. In the theoretical calculation part, different atomic layer numbers are used to represent the different modulation ratios of nano-multilayers, which has been reflected in other papers [44] in which the TiAlN/ZrN nano-multilayer was investigated and the most stable interface was found at *t*_TiAlN_:*t*_ZrN_ = 1:4. The difference for this work with the report of reference [44] in the modulation ratio of the most stable interface is due to the difference in the lattice structure and the interfacial bonding for different elements in the interface.

**Figure 9:**
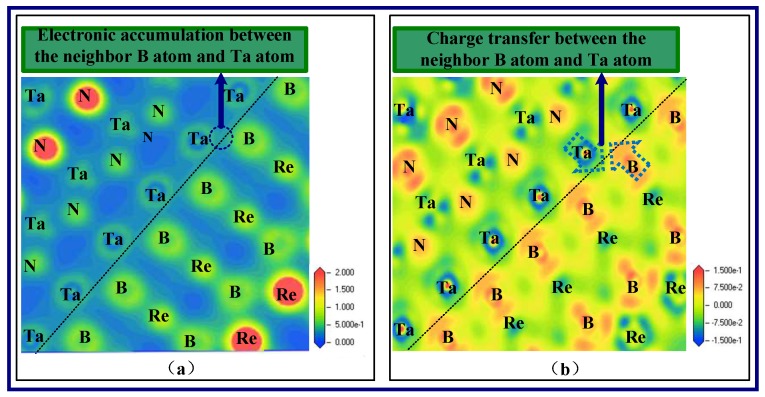
Charge density of 9ReB_2_/21TaN interface (**a**) and charge density difference of 9ReB_2_/21TaN interface (**b**), respectively, based on five B-Ta interfacial models.

To further clarify the behavior of the B-Ta interface with the 9ReB_2_/21TaN structure, the charge densities, which express the spatial distribution of electrons in the system, and the charge density differences, which express the relative electron transfer for each atom during the construction of the interface system, are calculated. The charge density can be available directly, and the charge density difference is given in refs [45,46,47,48]:

(7) $$\Delta \rho_{{\mathrm{ReB}}_{2}/\mathrm{TaN}}=\rho_{total}-\rho_{{\mathrm{ReB}}_{2}}-\rho_{\mathrm{TaN}}$$

where *ρ_total_* is the total charge density of the ReB_2_/TaN interface systems, and *ρ*_ReB2_ and *ρ*_TaN_ are the charge densities for the ReB_2_ and TaN relaxed isolated slabs, respectively. The charge densities and the charge density differences in the interfaces are presented in Figure 9a,b, respectively. The short dashed line represents the interfaces because the selected plane can pass through the interfacial B and Ta atoms and directly provide the bonding interactions between them. From Figure 9a, due to the charge distributions in ReB_2_ and TaN, the chemical bonding, which presents the covalent properties, can be found between the neighboring B and Ta atoms. Moreover, from Figure 9b, the charge transfer between the neighboring B and Ta atoms can be observed in which more blue parts means more electrons that the atoms loses, and more red parts means more electrons that the atoms receive. This indicates that some electrons transfer from Ta atoms to B atoms during the interface building, thereby forming ionic bonds between them. From the results, the chemical bonding between the neighboring B and Ta atoms in the interface system shows both covalency and iconicity.

**Figure 10:**
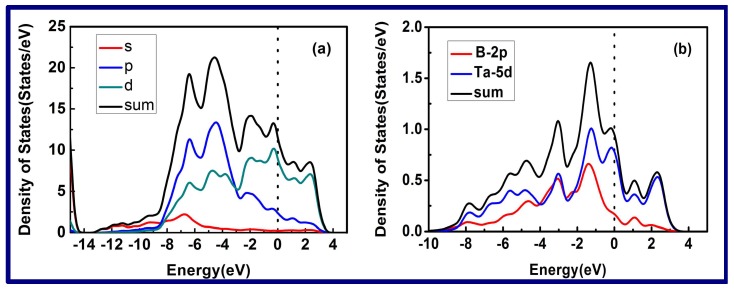
Density of states (DOS) of 9ReB_2_/21TaN interface (**a**) and interface atoms of 9ReB_2_/21TaN interface (**b**), respectively, based on five B-Ta interfacial models.

For a more detailed understanding of the entire interfacial interactions, the density of states (DOS) for the entire B-Ta interface with the 9ReB_2_/21TaN structure is calculated, as shown in Figure 10a. The DOS of the B-Ta interface shows that the wave overlaps the Fermi level, which demonstrates that the interface exhibits metallic characteristics. It is clear that the largest contribution to the total DOS is the d and p orbital electrons, while the s orbital electrons’ contribution is relatively small. In Figure 10b, we calculate the density of states (DOS) of two interface linking atoms for the B-Ta interface in order to understand the interfacial interatomic influence. It is clear that the largest contribution to the interfacial DOS for the B1-Ta interface is the B-2p and Ta-5d orbitals. In addition, the covalency bonding also exists between the B and Ta atoms in the interface due to the electron orbitals.

## 4. Conclusions

The ReB_2_/TaN multilayers are well suited for fundamental and application studies of protective coatings, given their superior mechanical properties and their clear modulated structure. The interfacial models are set up and the hardness mechanism and the relationship between the mechanical properties and the multilayered structure are explained from the calculations of the interfacial energy, adsorption energy, charge density, and density of states (DOS) using first-principles based on density functional theory (DFT). The following conclusions are made:

(1) The microstructure evolutions in the ReB_2_/TaN multilayers are carefully investigated by varying the modulation periods and modulation ratios. Clear coherent interfacial structures form between epitaxial layers at the optimal modulation period of 10 nm and the modulation ratio of 1:1. The fine nanocrystallites with small grain sizes are kept stable in individual layers at the optimal modulation condition.
(2) A maximum hardness of 38.7 GPa occurs at *Λ* = 10 nm and *t*_ReB2_:*t*_TaN_ = 1:1. The highest multilayer also displays the highest fracture resistance and the highest resistance to plastic deformation.
(3) The shallow indentations show little difference in hardness between the monolithic coatings and the multilayers. However, variations in the load versus displacement curves are observed at deeper indentation depths, indicating an enhancement of the hardness. One can deduce that the interface has a strong influence on the increase in the hardness.
(4) Six possible multilayered interfaces, B-N, BB-N, Re-N, B-Ta, BB-Ta, and Re-Ta, including eighteen interface configurations of top, hcp, and bridge, are established. The highest adsorption energy, hence the best interface stability, occurs in the B-Ta interface configuration. The strengthening mechanisms of the multilayered structure are elucidated using the calculation results of the interfacial energies to advance the understanding of the relationship between the superior mechanical properties and the interfacial structure.
(5) The 3ReB_2_/27TaN, 9ReB_2_/21TaN, 15ReB_2_/15TaN, 21ReB_2_/3TaN, and 27ReB_2_/3TaN interfacial models are established to further explain the underlying mechanism for why the residual stress depends on the interfacial stability. The multilayers at a *t*_ReB2_:*t*_TaN_ of 1:3 exhibits the lowest residual stress, which agrees with the lowest interfacial energy and the highest adsorption energy of the 9ReB_2_/21TaN interfacial structure. Therefore, the 9ReB_2_/21TaN interfacial configuration is found to be the most stable interface, which is a main contribution to the residual stress release.

## Figures and Tables

**Table 1:** The values of *H*^3^/*E*^2^ for the multilayers (*H*^3^/*E*^2^ of monolithic ReB_2_ and TaN coatings is 11.9% and 4.5%).

*Λ* (nm)	*t*_ReB2_:*t*_TaN_	*H*^3^/*E*^2^ (%)
4	1:1	4.35
10	1:1	25.8
30	1:1	4.09
10	5:1	4.89
10	1:2	11.5

**Table 2:** The adsorption energy (*E_ad_*) corresponding to eighteen interfacial models.

Stacking	*E_ad_* (J/m^2^)	Stacking	*E_ad_* (J/m^2^)	Stacking	*E_ad_* (J/m^2^)
B1-N hcp	−2.668	B1-N top	−3.244	B1-N bridge	−3.301
B2-N hcp	−2.668	B2-N top	−2.653	B2-N bridge	−2.665
Re-N hcp	0.145	Re-N top	0.063	Re-N bridge	1.110
B1-Ta hcp	6.341	B1-Ta top	6.028	B1-Ta bridge	7.046
B2-Ta hcp	6.316	B2-Ta top	6.296	B2-Ta bridge	7.213
Re-Ta hcp	4.814	Re-Ta top	4.090	Re-Ta bridge	5.142
